# Health-Related Quality of Life and Experiences of Brazilian Celiac Individuals over the Course of the Sars-Cov-2 Pandemic

**DOI:** 10.3390/nu13051582

**Published:** 2021-05-09

**Authors:** Ana Luísa Falcomer, Priscila Farage, Cláudia B. Pratesi, Riccardo Pratesi, Lenora Gandolfi, Eduardo Yoshio Nakano, António Raposo, Renata Puppin Zandonadi

**Affiliations:** 1 Department of Nutrition, School of Health Sciences, University of Brasilia, 70910-900 Brasilia, Brazil; anafalcomer@gmail.com; 2 Faculty of Nutrition, Federal University of Goias, 74605-080 Goiânia, Brazil; priscilafarage@ufg.br; 3 Interdisciplinary Laboratory of Biosciences and Celiac Disease Research Center, School of Medicine, University of Brasilia, 70910-900 Brasilia, Brazil; cpprates@eckerd.edu (C.B.P.); pratesiunb@gmail.com (R.P.); lenoragandolfi1@gmail.com (L.G.); 4 Department of Statistics, University of Brasilia, 70910-900 Brasilia, Brazil; eynakano@gmail.com; 5 CBIOS (Research Center for Biosciences and Health Technologies), Universidade Lusófona de Humanidades e Tecnologias, Campo Grande 376, 1749-024 Lisboa, Portugal

**Keywords:** celiac disease, quality of life, COVID-19, pandemic

## Abstract

Since the end of 2019, the world has been facing an unpredicted COVID-19 pandemic with consequences for the economy, environment, society, and health. The COVID-19 pandemic has increased the risk of death, bringing unbearable psychological pressure upon people worldwide. For celiac patients, the pandemic may represent an additional burden concerning the inherent aspects of celiac disease (CD) that compromise these individuals’ quality of life (QoL). Therefore, the objective of this study was to evaluate Brazilian celiac patients’ QoL during the course of the COVID-19 pandemic caused by its outbreak and rapid spread and subsequent restrictive measures in addition to the dietary restrictions and other burdens caused by CD. This country-wide cross-sectional study was conducted using a self-administered instrument previously validated in Brazilian–Portuguese to investigate the QoL of individuals with CD. Data collected through the online self-administration of the Brazilian version of the celiac disease quality of life questionnaire (CDQ) comprised 674 CD individuals’ responses. Although pandemics have historically posed a challenge for Brazilian population, this period was not associated with a negative impact on Brazilian CD individuals’ QoL. During the pandemic, the QoL of Brazilian’s with CD was more affected by gastrointestinal aspects than emotions and social aspects and worries. Gender, age, marital status, having (or not) children, occupation, and a positive test for COVID-19 did not affect CD individuals’ QoL. However, the study revealed a larger burden and diminished QoL for individuals not following a gluten-free diet and those using antidepressants. Additional research is necessary to verify how the length of the pandemic will affect celiac individuals and then compare those outcomes compare to the COVID-19 period and after.

## 1. Introduction

Celiac disease (CD) is a chronic enteropathy started by gluten ingestion in genetically susceptible individuals. CD affects approximately 1% of the world’s individuals and presents clinical manifestations including intestinal and extraintestinal symptoms [1]. Despite the symptoms and consequences of gluten ingestion, which are directly related to how these patients perceive their quality of life (QoL) [2], following a strict gluten-free diet (GFD) can also affect QoL positively (reducing symptoms) [3] or negatively (due to the social exclusion, lack of information and ability to handle healthy gluten-free meal production, the high price of food, risk of gluten cross-contamination, fear of social exclusion, insecurity of being safe, and others) [4]. Therefore, diet is one of the most important aspects of the QoL of CD individuals.

The world is facing an unpredicted COVID-19 pandemic generated by Sars-CoV-2 viruses since the end of 2019. COVID-19 has brought consequences upon the economy, society, environment, and health. Worldwide, people are experiencing an increase in the risk of death and unbearable psychological burden [5]. For celiac patients, the pandemic may represent additional burdens concerning CD’s inherent aspects that compromise these individuals’ QoL. As a result, celiac individuals may perceive their QoL affected in several ways including physical, emotional, economic, and social [3,6,7]. Most governments around the world took severe mitigation measures due to the COVID-19 pandemic, including community-wide lockdowns, home quarantines, social distancing, and the prohibition of social gatherings, to reduce the spread of Sars-CoV-2 [8]. These measures limited people’s access to traditional medical routine appointments and access to food from food services and markets, which may represent an even worse disturbance for individuals who present dietary restrictions.

A recent study conducted in Poland with 1033 adults investigated perceived stress as a predictor of consumers’ distress of restricted access to food and predictor of food purchase behaviors throughout the pandemic [9]. The study showed that more than half of the participants perceived a reduction in grocery stores’ food supplies. The fear experienced during the pandemic is also influenced by changes in food availability, which highlights the importance of available information and confidence in their sources to reduce psychological burden [9]. Hence, we hypothesized that the Sars-Cov-2 pandemic could affect groups that present chronic diseases whose treatment and well-being depend on restricting diet and special foods, like CD, leading to influences on their food security [10] and, consequently, in their QoL.

Therefore, the investigation of celiac patients’ health-related QoL during the Sars-Cov-2 pandemic is essential, since they have special dietary needs. Furthermore, CD interferes in the patient’s daily life beyond the gastrointestinal and health aspects of the disease such as their social, economic, and emotional status [11]. While several studies have been carried out on the pandemic’s psychological effects on the general public, patients, medical staff, children, and elderly [5,12,13,14], and several other studies were performed on celiac individuals during the pandemic [15,16,17,18,19,20,21,22], none evaluated the QoL of CD patients during the difficult times of the pandemic. Therefore, the objective of this research was to evaluate Brazilian celiac patients’ QoL during the pandemic caused by the outbreak, rapid spread, and subsequent restrictive measures caused by COVID-19, in addition to the dietary restrictions and other burdens caused by CD. We aimed to show what influences Brazilian celiac patients’ QoL during the pandemic, helping them recover after the pandemic period.

## 2. Materials and Methods

### 2.1. Study Design and Instrument

This country-wide cross-sectional research was performed using a self-administered instrument to evaluate a CD individual’s quality of life (CDQ) developed by Häuser et al. [23], which was validated in Brazil by Pratesi et al. [6], to investigate the QoL of Brazilian celiac individuals during the pandemic. The CDQ comprises four domains (i.e., emotions, gastrointestinal symptoms, concerns, and social) with 7 items each, comprising a 28-item questionnaire. Each question was evaluated using a 7 point scale (“1” meaning the worst QoL perception and “7” the best QoL perception). Therefore, the highest possible final score for QoL was 196 points, with higher scores reflecting a better level of QoL.

Sociodemographic characteristics were also investigated in this study (e.g., gender, age, marital status, place of residency, educational level, occupation). The GFD adhesion was self-reported using the Brazilian version of the instrument previously validated [6], and it was not confirmed by serological tests since, during the pandemic, we could not access patients. The researchers also included questions on the Sars-CoV-2 period regarding the presence of a positive test for the disease and/or a family member with infection.

The SurveyMonkey® platform was used for CDQ applications from 7 to 28 August 2020. Volunteers were recruited nationwide by invitation through a link to access the study sent via email, messaging apps, Brazilian Celiac Associations, and social networks.

### 2.2. Participants and Ethics

A convenience sample composed of CD individuals from the entire country was used in this study. The participants were recruited through a research link invitation that included a consent form to certify their agreement to join the study. The research was approved by the Ethics Committee of the University of Brasília (CAEE 69119317.3.0000.0030) and conducted according to the Declaration of Helsinki guidelines. 

The inclusion criteria were: (i) to have celiac disease; (ii) live in Brazil; (iii) ≥18 years old. Individuals who agreed to participate in the research were directed to the survey’s questions, while those who did not agree to participate were directed to a page thanking them for their time.

### 2.3. Statistical Analysis

Data from the SurveyMonkey® platform were extracted and analyzed using the IBM SPSS Statistics for Windows (Armonk, NY: IBM Corp, USA). The statistical analysis was conducted by scores (higher score indicating a higher QoL). The corresponding dimensions’ median value substituted blank questions. The total score was calculated for each individuals’ characteristics. If there was more than 20% of blank questions, the questionnaire was not used in the analysis.

Descriptive statistics were presented as the mean, median, standard deviation, and floor and ceiling effect of CDQ’s subscales. One-way repeated measures ANOVA followed by Bonferroni’s post-hoc test were used to compare domains’ means. The Student’s *t*-test and one-way analysis of variance (ANOVA) followed by Tukey’s post-hoc tests were used to compare the domains with the interesting variables. Comparisons of QoL before and during the Sars-Cov-2 pandemic were performed by one-way analysis of covariance (ANCOVA), considering the sociodemographic and health variables as controlling covariates. All tests considered bilateral hypotheses and a significance level of 5%. The factor validity was verified using a confirmatory factor analysis. The Chi-squared test of minimum discrepancy (chi2), the root mean square error of approximation (RMSEA), and the comparative fit index (CFI) evaluated the factor validity [24]. A RMSEA ≤ 0.05 and CFI ≥ 0.9 indicate a good model fit [25].

## 3. Results

### 3.1. Characterization of the Sample

Data collected through the online self-administration of the CDQ comprised responses from a convenience sample of 674 CD individuals. Most participants were female (*n* = 631; 93.6%), aged 39 years old or less (*n* = 405; 60.1%). CD diagnosis occurred before or at 39 years old for most participants (*n* = 512; 75.9%). Other socioeconomic, demographic, and health-related variables were also investigated, and the results are presented in Supplementary Materials Table S1. During the study period, 59.1% of the participants (*n* = 397) reported not having a partner compared to 40.9% of those with a partner (*n* = 275). Individuals were also asked whether they lived with children who were under 18 years of age, and most reported that they did not (57.7%; *n* = 387). Our sample was mainly composed of participants with a higher level of education, college-level and above. A total of 45.5% reported having a postgraduate degree; 38.3% went to college; and the other 16.2% attended high school, at most. In regard to occupation, 51.2% worked as a self-employed professional or in a private company (*n* = 335), 23.4% worked in public agencies (*n* = 153), 12.2% were students (*n* = 80), 13.1% were retired or unemployed (*n* = 86), and the remaining participants did not answer this question *n* = 20).

Self-reported adherence to the gluten-free diet was also investigated. Most participants (88.6%; *n* = 597) reported that they always followed the diet, while 11.4% (*n* = 77) reported not always sticking to the CD’s dietary restrictions. Concerning the use of antidepressants, most of the sample (78.4%; *n* = 526) did not take this type of medication. Regarding the specific questions about COVID-19, most participants (93.8%; *n* = 631) had nether tested positive for the infection up to the questionnaire’s completion date nor had an infected family member (65.9%; *n* = 444). Among those with family members who tested positive for the disease (34.1%; *n* = 230), most did not live together (77.8%; *n* = 179).

### 3.2. Instrument Reliability Analysis

The questionnaire’s and subscales’ internal consistencies were assessed through the Cronbach’s alpha measure (Table 1). The CDQ questionnaire’s four domains displayed satisfactory results for Cronbach’s alpha (α > 0.7), indicating the instrument’s good reliability, both separately by domain and as the complete instrument. Each domain’s scores ranged from 7 to 49, and the total from 28 to 196. Higher scores reflected a better level of QoL.

### 3.3. Quality of Life

The CDQ sub-scores found in this study were subcategorized by sociodemographic data and are presented in Table 2. In general, during the pandemic, the CDQ total score was affected by the age of CD diagnosis, educational level, GDF adhesion, and use of antidepressants.

There was neither a significant difference for the overall QoL score nor for each domain separately regarding gender. Concerning the variable “age”, the only significant difference observed was found for the domain “emotions”, which presented a lower score for individuals aged 39 years or less. The CD diagnosis age was reflected on the total score of QoL (*p* = 0.006) and was higher for those ≥40 years of age when they were diagnosed. Individuals diagnosed at ≤39 years of age displayed lower QoL overall scores and in the “gastrointestinal” and “emotions” domains.

The absence of a partner led to higher scores for the “gastrointestinal” and “emotions” domains in comparison to the presence of a partner (*p* = 0.021 and *p* = 0.001, respectively), but it did not reflect significantly better overall QoL (*p* = 0.264). Children under 18 years old and living in the same house as the participant resulted in a significantly lower score only for the “worries” domain (*p* = 0.009).

In general, a greater educational level was associated with higher global QoL and higher scores for the domains separately. Regarding the type of occupation, a significant difference was only found for the “worries” domain. Students displayed higher scores for this domain in comparison to retired/unemployed participants.

Adherence to the gluten-free diet—represented by participants’ disclosure of “always following the diet”—resulted both in higher overall QoL and higher scores for each domain separately. Concerning antidepressants, participants who took this type of medication showed lower overall QoL and lower scores for each domain individually. 

No significant difference in the quality of life was found between participants who had COVID-19 and those who did not. Moreover, having a relative infected with Sars-CoV-2 also did not significantly differ in the QoL.

## 4. Discussion

This is the first study on CD individuals’ QoL performed in Brazil during a pandemic. Identifying the factors influencing the CD individuals’ QoL during the pandemic could help design strategies to mitigate them. Our results showed that most participants were female (*n* = 631; 93.6%), similar to the study CD individuals’ QoL performed in Brazil before the pandemic in which 94% (*n* = 425) were female and 82.4% (*n* = 371) were not using antidepressants [6]. The age group (60.1%, ≤39 y) of the participants was also similar to the participants prior to the pandemic, aged 39 years old or less (57%, ≤39 y [6]). In our study and the previous one [6], 88.6% of participants reported following a strict GFD. Since our sample of CD individuals presented characteristics regarding gender, age, and GFD adherence that were similar, we compared our QoL data to the previous study performed using the same QoL questionnaire in Brazil [6] (Supplementary Materials Table S2). The comparison among the groups of Brazilian celiac individuals before and during the pandemic was possible since, despite it being impossible to identify if they were the same individuals, they were from the same population under study and with similar characteristics. In addition, it was corrected using analysis of covariance (ANCOVA) to adjust for possible heterogeneities in the socio-demographic characteristics of the samples from the two studies.

Surprisingly, the results obtained during the pandemic showed a higher total score for the CDQ than the previous application of the instrument in Brazil before the pandemic (*p* = 0.011). Therefore, our hypothesis that the pandemic could affect Brazilian CD individuals’ QoL was not confirmed. Analyzing each domain separately, a statistically higher score was found for “social” and “worries”(*p* < 0.001 in both cases) during the pandemic than before. On the other hand, the “gastrointestinal” domain displayed a lower score during the pandemic (*p* < 0.001). Regarding the domain “emotions”, the score was not statistically different from the CDQ’s prior application (*p* = 0.082). In theory, staying at home favors time to “eat well and stay well”, following a strict GFD [18]. This could positively impact on the “worries” and “emotions” domains (potentially reduced by the lowered fear of consuming gluten-containing products) but have a worse effect in the “social” domain (as occurred in our study) due to the necessary isolation during the pandemic. The study on Brazilian QoL in general individuals also showed that the “social” domain was burdened by pandemic [26].

During the pandemic, the domains “social” and “worries” presented the best scores among the domains, which differed from the study performed before the pandemic in which the best scores were attributed to the “gastrointestinal” and “social” domains [6].

The “gastrointestinal” domain was worse during the pandemic. Although a similar percentage of respondents informed that they followed a strict GFD in both periods (Supplementary Materials Table S2), data from the “gastrointestinal” domain might indicate the possibility of non-intentional consumption of gluten. Considering that CD individuals that live without a partner presented better scores in the “gastrointestinal” domain (Table 2) than the ones who lived with partners (added to the need to be and eat at home), the presence of more than one adult can, potentially, increase the potential for gluten cross-contamination when one of them does not follow the GFD.

Individuals who reported following the GFD presented better QoL overall (and by domains) than those who reported not adhering to a strict GFD. Similar results were found before the pandemic, except for the “worries” domain, which did not differ among individuals who followed the GFD [6]. Probably, people eating more often at home may reduce the perception of the risk of gluten ingestion for those following a strict GFD, and it could have impacted the “worries” domain. 

Different from the study that was performed with the general Brazilian population (*n* = 1877) during the pandemic in which gender differed for all QoL domains (males with better QoL than females) [26], no significant difference was found for the CDQ during the pandemic regarding gender. Our result was also different from the Brazilian CD individuals’ QoL study performed before the pandemic in which males’ scores for the CDQ were higher than females, except for the “gastrointestinal” domain. We did not expect the results in which gender, age, and marital status did not differ among the CD individuals’ perception of QoL during the pandemic, since previous studies with non-celiac individuals showed that younger and females individuals were at a greater risk for distress and that individuals with a partner showed better scores than the those with no partner [27,28,29,30].

The individuals in the older age group handled the “emotions” domain better in this pandemic than the younger age group. This result is aligned with other studies with non-celiac individuals during the pandemic [31,32]. The authors attributed their results to more unsure working conditions and economic burdens for younger people and larger restrictions for younger than for older individuals [31,32]. However, in our study, the “social” domain did not differ between age groups. It is important to mention that the previous Brazilian CD individuals’ QoL study [6] showed that prompt diagnosis was related to a better “social” domain score and general QoL, similar to other studies [33,34]. However, in this study, the time of CD diagnosis did not influence QoL (Pearson correlation = 0.1).

Individuals with the highest educational level presented better overall QoL and higher scores for the domains separately, as found in the study performed in CD individuals’ QoL in Brazil before the pandemic [6]. This is probably because a higher educational level contributes to an individual’s physical, social, and health aspects. Low education increases some chronic health conditions’ adverse outcomes because of the low level of knowledge [35,36,37,38]. Education level tends to be associated with higher socioeconomic status [36], and income modulates health-seeking behavior and access to health care [39], both related to higher QoL. In this sense, it is well-known that higher education levels can influence some of the QoL aspects. This characteristic of our convenience sample could be a potential bias of our study. Additional studies should be performed to evaluate CD individuals with lower education levels and their influence on individuals’ QoL.

Our results showed that 6% (*n* = 42) of CD individuals experienced COVID-19 as did 34% (*n* = 230) of their relatives. These results were higher than those found in a study [20] conducted with 138 CD individuals in Italy in April/May 2020, where no diagnosis of COVID-19 were reported, while 19 participants presented flu-like symptoms (one having a negative nasopharyngeal swab test). Further, 7.9% (*n* = 11) CD individuals reported a relative presenting respiratory symptoms suggestive of Sard-CoV-2 infection [20]. Our findings were worse compared to the study with the general Brazilian population [26] in which 2.7% of the participants had COVID-19 and 16% (*n* = 300) of the participants had a relative who presented Sars-Cov-2 infection. Considering that the Brazilian study with the general population ended data collection (14 August 2020) 14 days before our study (28 August 2020) and the number of cases and deaths were continuously increasing, it is not possible to affirm that the incidence of COVID-19 was higher in CD individuals or their relatives than the general population in Brazil. As only 42 patients (6.2%) had SARS-CoV-2 infection when the questionnaire was completed, differences in QoL perception were not likely significant. 

A large study performed with 10,737 CD patients from Argentina, Australia, Canada, Italy, Mexico, New Zealand, Spain, Uruguay, and the United States during the pandemic showed that CD patients have similar chances of contracting Sars-CoV-2, and it is unnecessary to take additional care to prevent exposure aside from the recommendations to the general population [22]. However, the comorbidities identified as a significant predictor of morbidity and mortality on COVID-19 were more frequent in CD individuals than control ones [22]. A cohort study conducted in Sweden with 40,963 CD individuals showed that they were neither at increased risk of hospitalization for COVID-19 than the control ones nor at high risk for severe disease outcomes and mortality [21].

Individuals that contracted or did not contract COVID-19 did not differ in QoL. The same was observed among participants whose relatives contracted or did not contract COVID-19, probably because the uncertainties about the disease and its outcomes lead people to continue to fear infection by the virus [40,41], despite having been contaminated themselves or one of their relatives.

A potential study bias is the method of survey dissemination (i.e., internet, email, and social media) and the use of a convenience sample. However, if random sampling was used, it would be impossible to reach a large sample. Also, during the pandemic period and social isolation, use of the internet is the primary way to reach respondents. Despite similar characteristics between the samples of the two studies conducted on CD individuals’ QoL in Brazil, it was not possible to affirm that the same individuals participated in both studies. As the vast majority of the respondents to the survey were female celiac subjects, the results did not reflect the perceptions of the male population.

## 5. Conclusions

The COVID-19 pandemic poses a historic challenge for many worldwide, but this period has not been associated with a negative impact on the Brazilian CD individuals’ QoL. In the course of the pandemic, CD individuals’ QoL was more affected by gastrointestinal and social aspects than emotional aspects and worries in Brazil. Gender, age, marital status, having (or not) children, occupation, and a positive COVID-19 test did not affect the CD individuals’ QoL. However, the study revealed a major QoL burden for individuals who did not follow the GFD and for those using antidepressants. Additional research is necessary to verify how the length of the pandemic will affect celiac individuals and then to compare this period during and after COVID-19.

## Figures and Tables

**Table 1:** Analysis of the precision of the subscales (*n* = 674) of the CDQ instrument.

	Mean (SD)	Median (IQR)	Range	Floor Effect (%)	Ceiling Effect (%)	Internal Consistency (Cronbach’ Alpha)
Emotion	34.81 (8.42)	35 (29–42)	10–49	0%	2.1%	0.820
Social	25.82 (8.87)	26 (19–32)	7–49	0.9%	0.1%	0.916
Worries	34.86 (10.25)	36 (27–44)	8–49	0%	6.1%	0.842
Gastrointestinal	29.77 (10.75)	30 (21–39)	7–49	0.6%	1.5%	0.838
Total Score	125.26 (32.02)	127 (100–151)	43–194	0%	0%	0.936

**Table 2:** CDQ sub-scores subcategorized by sociodemographic data (*n* = 674).

	Gastrointestinal		Emotions		Social		Worries		Total	
	Mean (SD)	*p*	Mean (SD)	*p*	Mean (SD)	*p*	Mean (SD)	*p*	Mean (SD)	*p*
Gender *										
Women (*n* = 631)	34.87 (8.46) ^a^	0.534	25.87 (8.75) ^a^	0.663	34.85 (10.28) ^a^	0.925	29.70 (10.82) ^a^	0.548	125.29 (32.07) ^a^	0.940
Men (*n* = 43)	34.05 (7.72) ^a^		25.14 (10.59) ^a^		35.00 (9.77) ^a^		30.72 (9.72) ^a^		124.91 (31.63) ^a^	
Age *										
≤39 (*n* = 405)	34.34 (8.54) ^a^	0.090	24.66 (8.54) ^a^	<0.001	34.61 (10.42) ^a^	0.463	29.49 (10.83) ^a^	0.442	123.11 (32.01) ^a^	0.039
≥40 (*n* = 264)	35.47 (8.20) ^a^		27.51 (9.11) ^b^		35.21 (10.02) ^a^		30.14 (10.56) ^a^		128.34 (31.75) ^a^	
Age at diagnosis *										
≤39 (*n* = 512)	34.46 (8.46) ^a^	0.049	25.02 (8.59) ^a^	<0.001	34.46 (10.35) ^a^	0.071	29.40 (10.91) ^a^	0.119	123.34 (32.00) ^a^	0.006
≥40 (*n* = 162)	35.94 (8.21) ^b^		28.35 (9.29) ^b^		36.12 (9.81) ^a^		30.91 (10.17) ^a^		131.33 (31.43) ^b^	
										
Marital status *										
No partner (*n* = 397)	35.46 (8.46) ^a^	0.021	26.77 (8.95) ^a^	0.001	34.98 (10.19) ^a^	0.803	29.30 (10.73) ^a^	0.148	126.52 (32.39) ^a^	0.264
With partner (*n* = 275)	33.94 (8.29) ^b^		24.47 (8.62) ^b^		34.78 (10.29) ^a^		30.52 (10.75) ^a^		123.72 (31.42) ^a^	
Children under 18*										
Yes (*n* = 284)	34.89 (8.53) ^a^	0.805	25.75 (8.65) ^a^	0.856	34.14 (10.25) ^a^	0.122	28.46 (10.60) ^a^	0.009	123.25 (31.65) ^a^	0.174
No (*n* = 387)	34.73 (8.36) ^a^		25.88 (9.07) ^a^		35.39 (10.24) ^a^		30.66 (10.78) ^b^		126.66 (32.31) ^a^	
Educational level **										
≤High school (*n* = 109)	32.90 (8.98) ^a^		23.96 (9.11) ^a^		32.59 (9.99) ^a^		27.09 (10.98) ^a^		116.54 (32.38) ^a^	
College (*n* = 258)	33.36 (8.57) ^a^	<0.001	25.20 (8.77) ^ab^	0.003	33.61 (10.39) ^a^	<0.001	28.71 (10.54) ^a^	<0.001	120.87 (31.72) ^a^	<0.001
Postgraduate degree (*n* = 307)	36.73 (7.67) ^b^		27.00 (8.73) ^b^		36.71 (9.92) ^b^		31.61 (10.55) ^b^		132.05 (30.83) ^b^	
Occupation **										
Self-employed/private company (*n* = 335)	35.18 (8.06) ^a^		25.95 (8.93) ^a^		35.51 (9.95) ^a^		30.35 (10.67) ^ab^		126.99 (31.21) ^a^	
Public agency (*n* = 153)	35.39 (8.54) ^a^	0.104	26.86 (8.59) ^a^	0.076	34.57 (10.44) ^a^	0.114	29.28 (11.14) ^ab^	0.044	126.10 (33.01) ^a^	0.084
Student (*n* = 80)	33.00 (9.26) ^a^		24.56 (8.32) ^a^		34.91 (9.17) ^a^		30.66 (11.27) ^a^		123.14 (31.86) ^a^	
Retired/unemployed (*n* = 86)	33.85 (8.69) ^a^		24.10 (9.71) ^a^		32.53 (11.42) ^a^		26.86 (9.63) ^b^		117.35 (32.90) ^a^	
Gluten-free diet *										
Always (*n* = 597)	35.48 (8.36) ^a^	<0.001	26.40 (8.77) ^a^	<0.001	35.20 (10.26) ^a^	0.015	30.38 (10.66) ^a^	<0.001	127.46 (31.78) ^a^	<0.001
Not always (*n* = 77)	29.71 (7.03) ^b^		21.29 (8.35) ^b^		32.18 (9.79) ^b^		25.03 (10.32) ^b^		108.21 (28.77) ^b^	
Antidepressants *										
Yes (*n* = 145)	32.46 (8.37) ^a^	<0.001	22.15 (7.61) ^a^	<0.001	33.28 (10.05) ^a^	0.032	27.70 (10.70) ^a^	0.008	115.59 (30.27) ^a^	<0.001
No (*n* = 526)	35.51 (8.30) ^b^		26.86 (8.94) ^b^		35.35 (10.25) ^b^		30.36 (10.69) ^b^		128.08 (31.94) ^b^	
Positive test for COVID-19 *										
Yes (*n* = 42)	34.38 (8.85) ^a^	0.723	26.71 (9.51) ^a^	0.509	36.98 (9.10) ^a^	0.131	31.24 (10.74) ^a^	0.367	129.31 (32.19) ^a^	0.405
No (*n* = 631)	34.86 (8.40) ^a^		25.78 (8.82) ^a^		34.73 (10.31) ^a^		29.69 (10.74) ^a^		125.06 (32.00) ^a^	
Infected Family Member **										
Yes: living together (*n* = 51)	34.86 (8.80) ^a^		27.80 (9.28) ^a^		35.69 (8.88) ^a^		31.96 (9.41) ^a^		130.31 (29.76) ^a^	
Yes: do not live together (*n* = 179)	34.02 (8.50) ^a^	0.328	25.08 (8.80) ^a^	0.147	34.40 (10.13) ^a^	0.695	28.77 (10.80) ^a^	0.152	122.26 (32.18) ^a^	0.222
No (*n* = 444)	35.14 (8.33) ^a^		25.89 (8.83) ^a^		34.95 (10.45) ^a^		29.92 (10.85) ^a^		125.89 (32.16) ^a^	

Some variables do not add up to 674, since some individuals did not fill in all fields. * Student’s *t*-test. ** ANOVA with Tukey’s post-hoc tests. Groups with the same letters do not differ significantly.

## Data Availability

The study did not report any data.

## Supplementary Materials

The following are available online at https://www.mdpi.com/article/10.3390/nu13051582/s1. Table S1: Socioeconomic, demographic, and health-related results of the study sample; Table S2: Comparison between the application of the CDQoL questionnaire before and during the Sars-Cov-2 pandemic.

## References

[1] Biagi F., Corazza G.R. (2010). Mortality in celiac disease. Nat. Rev. Gastroenterol. Hepatol..

[2] Casellas L.R.F., Rodrigo L., Vivancos S.R.P., Riestra S., Pantiga C., Baudet J.S., Junquera F., Diví V.P., Abadia C., Papo M. (2008). Factors that impact health-related quality of life in adults with celiac disease: A multicenter study. World J. Gastroenterol..

[3] Skjerning H., Hourihane J., Husby S., DunnGalvin A. (2017). A comprehensive questionnaire for the assessment of health-related quality of life in coeliac disease (CDQL). Qual. Life Res..

[4] Farage P., Zandonadi R.P. (2014). The Gluten-Free Diet: Difficulties Celiac Disease Patients have to Face Daily. Austin. J. Nutr. Food Sci..

[5] Cao W., Fang Z., Hou G., Han M., Xu X., Dong J., Zheng J. (2020). The psychological impact of the COVID-19 epidemic on college students in China. Psychiatry Res..

[6] Pratesi C., Häuser W., Uenishi R., Selleski N., Nakano E., Gandolfi L., Pratesi R., Zandonadi R. (2018). Quality of Life of Celiac Patients in Brazil: Questionnaire Translation, Cultural Adaptation and Validation. Nutrients.

[7] Häuser W., Stallmach A., Caspary W.F., Stein J. (2007). Predictors of reduced health-related quality of life in adults with coeliac disease. Aliment. Pharmacol. Ther..

[8] Wang X., Lei S.M., Le S., Yang Y., Zhang B., Yao W., Gao Z., Cheng S. (2020). Bidirectional Influence of the COVID-19 Pandemic Lockdowns on Health Behaviors and Quality of Life among Chinese Adults. Int. J. Environ. Res. Public Health.

[9] Jeżewska-Zychowicz M., Plichta M., Królak M. (2020). Consumers’ Fears Regarding Food Availability and Purchasing Behaviors during the COVID-19 Pandemic: The Importance of Trust and Perceived Stress. Nutrients.

[10] Brown E., Das R., Brewer A.G., Martinez E., Bilaver L.A., Gupta R.S. (2020). Food Insecure and Allergic in a Pandemic: A Vulnerable Population. J. Allergy Clin. Immunol. Pract..

[11] Zysk W., Głąbska D., Guzek D. (2018). Social and emotional fears and worries influencing the quality of life of female celiac disease patients following a gluten-free diet. Nutrients.

[12] Chen Q., Liang M., Li Y., Guo J., Fei D., Wang L., He L., Sheng C., Cai Y., Li X. (2020). Mental health care for medical staff in China during the COVID-19 outbreak. Lancet Psychiatry.

[13] Yang Y., Li W., Zhang Q., Zhang L., Cheung T., Xiang Y.T. (2020). Mental health services for older adults in China during the COVID-19 outbreak. Lancet Psychiatry.

[14] Brusciano L., Gualtieri G., Gambardella C., Tolone S., Lucido F.S., del Genio G., Pellino G., Docimo L. (2020). When preserving life becomes imperative, quality of life is eclipsed! COVID-19 outbreak impacting patients with pelvic floor disorders undergoing pelvic floor rehabilitation. Br. J. Surg..

[15] Siniscalchi M., Zingone F., Savarino E.V., D’Odorico A., Ciacci C. (2020). COVID-19 pandemic perception in adults with celiac disease: An impulse to implement the use of telemedicine: COVID-19 and CeD. Dig. Liver Dis..

[16] Gokden Y., Hot S., Adas M., Koc D.O., Atak S., Hot A.B. (2020). Celiac disease and COVID-19 pandemic: Should we worry?. Acta Gastro Enterol. Belg..

[17] Monzani A., Lionetti E., Felici E., Fransos L., Azzolina D., Rabbone I., Catassi C. (2020). Adherence to the Gluten-Free Diet during the Lockdown for COVID-19 Pandemic: A Web-Based Survey of Italian Subjects with Celiac Disease. Nutrients.

[18] Elli L., Barisani D., Vaira V., Bardella M.T., Topa M., Vecchi M., Doneda L., Scricciolo A., Lombardo V., Roncoroni L. (2020). How to manage celiac disease and gluten-free diet during the COVID-19 era: Proposals from a tertiary referral center in a high-incidence scenario. BMC Gastroenterol..

[19] Nastro F.F., Tolone C., Serra M.R., Pacella D., Campanozzi A., Strisciuglio C. (2020). Prevalence of functional gastrointestinal disorders in children with celiac disease during the COVID-19 lockdown. Dig. Liver Dis..

[20] Zingone F., D’Odorico A., Lorenzon G., Marsilio I., Farinati F., Savarino E.V. (2020). Risk of COVID-19 in celiac disease patients. Autoimmun. Rev..

[21] Lebwohl B., Larsson E., Söderling J., Roelstraete B., Murray J.A., Green P.H., Ludvigsson J.F. (2021). Risk of Severe Covid-19 in Patients with Celiac Disease: A Population-Based Cohort Study. Clin. Epidemiol..

[22] Zhen J., Stefanolo J.P., Temprano M.D.L.P., Tedesco S., Seiler C., Caminero A.F., De-Madaria E., Huguet M.M., Vivas S., Niveloni S.I. (2021). The Risk of Contracting COVID-19 Is Not Increased in Patients With Celiac Disease. Clin. Gastroenterol. Hepatol..

[23] Häuser W., Gold J., Stein J., Caspary W.F., Stallmach A. (2006). Health-related quality of life in adult coeliac disease in Germany: Results of a national survey. Eur. J. Gastroenterol. Hepatol..

[24] Kline R. (2010). Principles and Practice of Structural Equation Modeling.

[25] Hu L., Bentler P. (1999). Cutoff criteria for fit indices in covariance structure analysis: Conventional criteria versus new alternatives. Struct. Equ. Model..

[26] Teotônio I., Hecht M., Castro L.C., Gandolfi L., Pratesi R., Nakano E.Y., Zandonadi R.P., Pratesi C.B. (2020). Repercussion of COVID-19 pandemic on Brazilians’ quality of life: A nationwide cross-sectional study. Int. J. Environ. Res. Public Health.

[27] Conversano C., Di Giuseppe M., Miccoli M., Ciacchini R., Gemignani A., Orrù G. (2020). Mindfulness, age and gender as protective factors against psychological distress during COVID-19 pandemic. Front. Psychol..

[28] Di Giuseppe M., Ciacchini R., Piarulli A., Nepa G., Conversano C. (2019). Mindfulness dispositions and defense style as positive responses to psychological distress in oncology professionals. Eur. J. Oncol. Nurs..

[29] Di Giuseppe M., Perry J.C., Conversano C., Gelo O.C.G., Gennaro A. (2020). Defense mechanisms, gender, and adaptiveness in emerging personality disorders in adolescent outpatients. J. Nerv. Ment. Dis..

[30] Carmassi C., Stratta P., Massimetti G., Bertelloni A.A., Conversano C., Cremone M.M., Miccoli M., Baggiani A., Rossi A., Dell’Osso L. (2014). New DSM-5 maladaptive symptoms in PTSD: Gender differences and correlations with mood spectrum symptoms in a sample of high school students following survival of an earthquake. Ann. Gen. Psychiatry.

[31] Pieh C., Budimir S., Probst T. (2020). The effect of age, gender, income, work, and physical activity on mental health during coronavirus disease (COVID-19) lockdown in Austria. J. Psychosom. Res..

[32] Ozamiz-Etxebarria N., Dosil-Santamaria M., Picaza-Gorrochategui M., Idoiaga-Mondragon N. (2020). Stress, anxiety, and depression levels in the initial stage of the COVID-19 outbreak in a population sample in the northern Spain. Cad. Saude Publica.

[33] Ramírez-Cervantes K.L., Remes-Troche J.M., del Pilar Milke-García M., Romero V., Uscanga L.F. (2015). Characteristics and factors related to quality of life in Mexican Mestizo patients with celiac disease. BMC Gastroenterol..

[34] Castilhos A.C., Gonçalves B.C., Macedo e Silva M., Lanoni L.A., Metzger L.R., Kotze L.M.S., Nishiara R.M. (2015). Quality of Life Evaluation in Celiac Patients From Southern Brazil. Arq. Gastroenterol..

[35] Gazmararian J.A., Williams M.V., Peel J., Baker D.W. (2003). Health literacy and knowledge of chronic disease. Patient Educ. Couns..

[36] Everson S.A., Maty S.C., Lynch J.W., Kaplan G.A. (2002). Epidemiologic evidence for the relation between socioeconomic status and depression, obesity, and diabetes. J. Psychosom. Res..

[37] Rojas-García A., Ruiz-Perez I., Rodríguez-Barranco M., Gonçalves Bradley D.C., Pastor-Moreno G., Ricci-Cabello I. (2015). Healthcare interventions for depression in low socioeconomic status populations: A systematic review and meta-analysis. Clin. Psychol. Rev..

[38] Elovainio M., Pulkki-Råback L., Jokela M., Kivimäki M., Hintsanen M., Hintsa T., Viikari J., Raitakari O.T., Keltikangas-Järvinen L. (2012). Socioeconomic status and the development of depressive symptoms from childhood to adulthood: A longitudinal analysis across 27 years of follow-up in the Young Finns study. Soc. Sci. Med..

[39] Mehra S., Leffler D.A., Pallav K., Tariq S., Shah S., Green P.H., Hansen J., Dennis M., Kelly C.P. (2011). Socioeconomic Status Influences Celiac Disease Diagnosis. Gastroenterology.

[40] Stojanov J., Malobabic M., Stanojevic G., Stevic M., Milosevic V., Stojanov A. (2020). Quality of sleep and health-related quality of life among health care professionals treating patients with coronavirus disease-19. Int. J. Soc. Psychiatry.

[41] Zhang Y., Ma Z.F. (2020). Impact of the COVID-19 pandemic on mental health and quality of life among local residents in Liaoning Province, China: A cross-sectional study. Int. J. Environ. Res. Public Health.

