# Supplementary material for: Health-Related Quality of Life and Experiences of Brazilian Celiac Individuals over the Course of the Sars-Cov-2 Pandemic
Source: Nutrients. 2021 May 9;13(5):1582. doi: 10.3390/nu13051582 (PMC8151969; doi:10.3390/nu13051582)
Supplement: Supplementary file 1 [file nutrients-13-01582-s001.zip › nutrients-1209320-supplementary.pdf]

Table S1. Socioeconomic, demographic, and health-related results of the study sample.

Gender	Age	Age at CD diagnosis	Marital status	Educational level	Occupation	Adherence to the GFD	Antidepressants	COVID-19	Children ≤18 y in the house	Family member with COVID-19
Women (*n*=631)<br>Men (*n*=43)	≤39 (*n*=405)<br>≥40 (*n*=264)	≤39 (*n*=512)<br>≥40 (*n*=162)	No partner (*n*=397)<br>With partner (*n*=275)	High school at most (*n*=109)<br>College degree (*n*=258)<br>Postgraduate degree (*n*=307)	Self-employed/private company (*n*=335)<br>Public agency (*n*=153)<br>Student (*n*=80)<br>Retired/unemployed (*n*=86)	Always (*n*=597)<br>Not always (*n*=77)	Yes (*n*=145)<br>No (*n*=526)	Yes (*n*=42)<br>No (*n*=631)	Yes (*n*=284)<br>No (*n*=387)	Yes: live together (*n*=51)<br>Yes: do not live together (*n*=179)<br>No (*n*=444)

* Not all variables add up to 674 responses, as some participants did not answer all the questions

Table S2. Comparison between the application of the CDQ QoL questionnaire before and during the Sars-Cov-2 pandemic.

	**Before COVID (*n* = 449) [1] Mean (SD) ***	**During COVID (*n* = 674) Mean (SD) ***	***p* ****
Emotions	26.66 (9.07)	25.69 (8.88)	0.082
Social	31.15 (8.43)	34.68 (8.26)	<0.001
Worries	27.22 (10.38)	34.67 (10.18)	<0.001
Gastrointestinal	34.77 (10.13)	29.65 (9.92)	<0.001
Total Score	119.80 (31.19)	124.68 (30.56)	0.011

* Estimated marginal means adjusted by gender, age, age at CD diagnosis, marital status, educational level, occupation, and adherence to the GFD and antidepressants.** One-way ANCOVA controlling by gender, age, age at CD diagnosis, marital status, educational level, occupation, adherence to the GFD, and antidepressants. $p < 0.05$ was considered statistically significant.

1. Pratesi, C.; Häuser, W.; Uenishi, R.; Selleski, N.; Nakano, E.; Gandolfi, L.; Pratesi, R.; Zandonadi, R. Quality of Life of Celiac Patients in Brazil: Questionnaire Translation, Cultural Adaptation and Validation. *Nutrients* **2018**, *10*, 1167, doi:10.3390/nu10091167.
